# Supplementary material for: EEG and behavioral correlates of attentional processing while walking and navigating naturalistic environments
Source: Sci Rep. 2021 Nov 16;11:22325. doi: 10.1038/s41598-021-01772-8 (PMC8595363; doi:10.1038/s41598-021-01772-8)
Supplement: Supplementary file 1 — Supplementary Information. [file 41598_2021_1772_MOESM1_ESM.docx]

Supplementary Materials

**EEG and behavioral correlates of attentional processing while walking and navigating naturalistic environments**

Magnus Liebherr^1,2*^, Andrew W. Corcoran^3,4*^, Phillip M. Alday^3,^, Scott Coussens^3^, Valeria Bellan^3,6^, Caitlin A. Howlett^3,6^, Maarten A. Immink^3,7^, Mark Kohler^3,8^, Matthias Schlesewsky^3^, Ina Bornkessel-Schlesewsky^3^

^1^Department of Neuroscience, Karolinska Institutet, Stockholm, Sweden; ^2^Department of General Psychology: Cognition, University Duisburg-Essen, Germany; ^3^Cognitive and Systems Neuroscience Research Hub, University of South Australia, Adelaide, Australia; ^4^Cognition and Philosophy Laboratory, Monash University, Melbourne, Australia; ^6^Innovation, Implementation & Clinical Translation (IIMPACT) in Health, University of South Australia, Adelaide, Australia; ^7^Sport, Health, Activity, Performance and Exercise Research Centre, Flinders University, Adelaide, Australia; ^8^School of Psychology, University of Adelaide, Adelaide, Australia

* Both authors contributed equally to the publication

Corresponding authors: Magnus Liebherr; Andrew W. Corcoran

Emails: magnus.liebherr@gmail.com; andrew.corcoran1@monash.edu

**
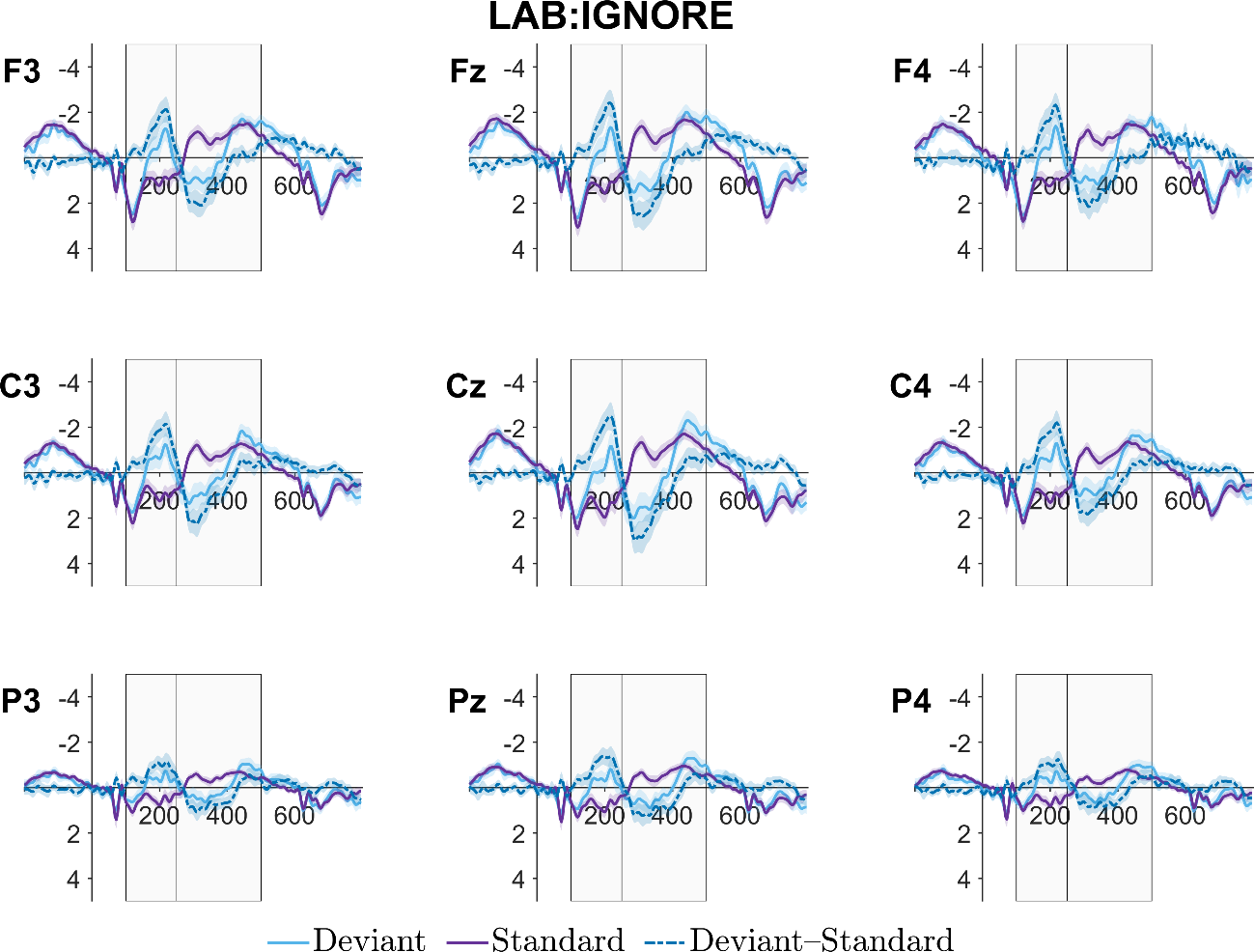
**

***Figure S1.*** Grand average event-related potentials (ERPs; solid lines) and difference waveforms (broken lines) from nine electrodes (F3, Fz, F4, C3, Cz, C4, P3, Pz, P4) during performance of the Ignore task in the Lab condition. Light blue waveforms depict ERPs evoked by deviant stimuli; purple waveforms depict ERPs evoked by standard stimuli. Broken lines indicate the difference in mean voltage (*μV*) between deviants and standards. Shading indicates the 84% confidence interval.

**
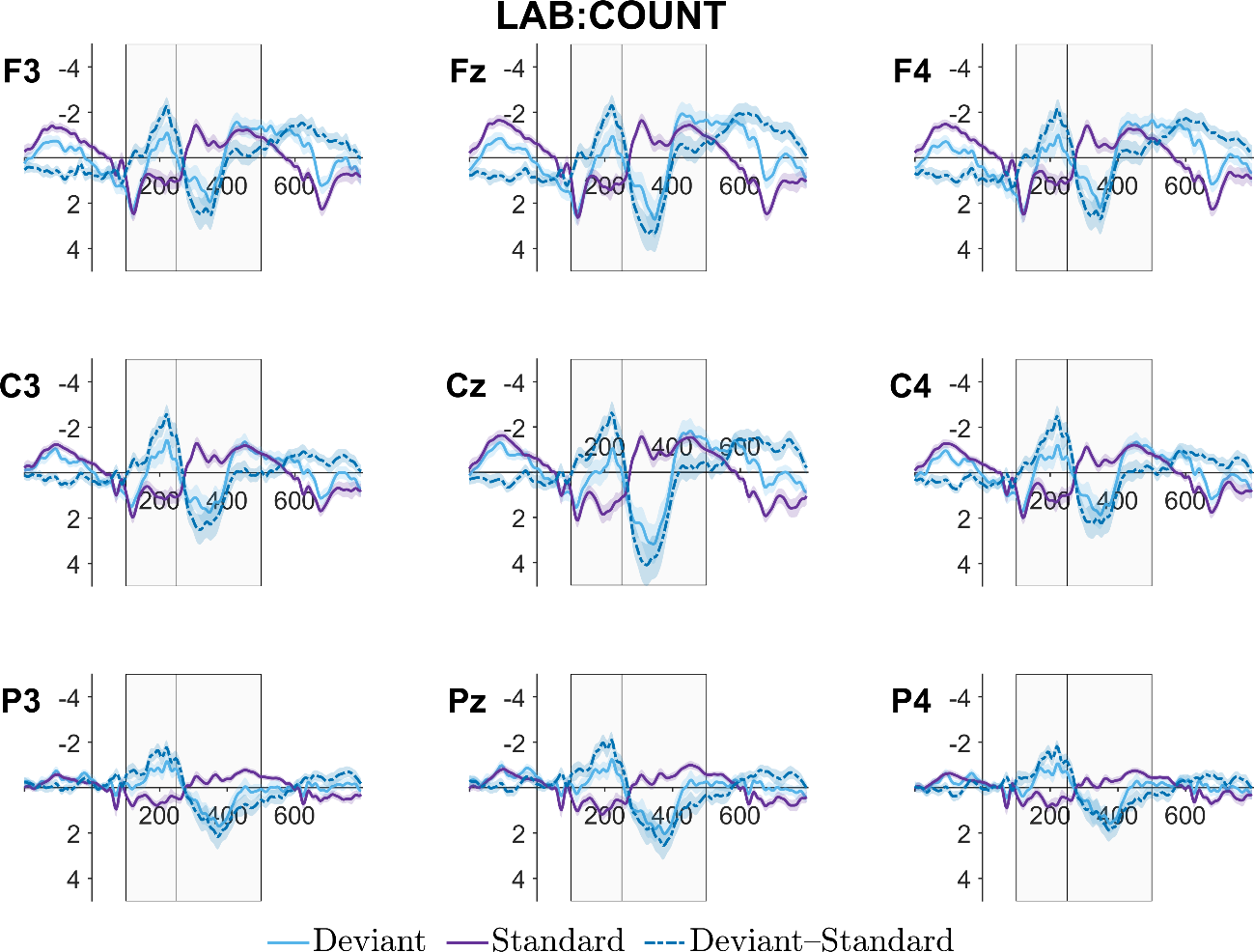
**

***Figure S2.*** Grand average event-related potentials (ERPs; solid lines) and difference waveforms (broken lines) from nine electrodes (F3, Fz, F4, C3, Cz, C4, P3, Pz, P4) during performance of the Count task in the Lab condition. Light blue waveforms depict ERPs evoked by deviant stimuli; purple waveforms depict ERPs evoked by standard stimuli. Broken lines indicate the difference in mean voltage (*μV*) between deviants and standards. Shading indicates the 84% confidence interval.

**
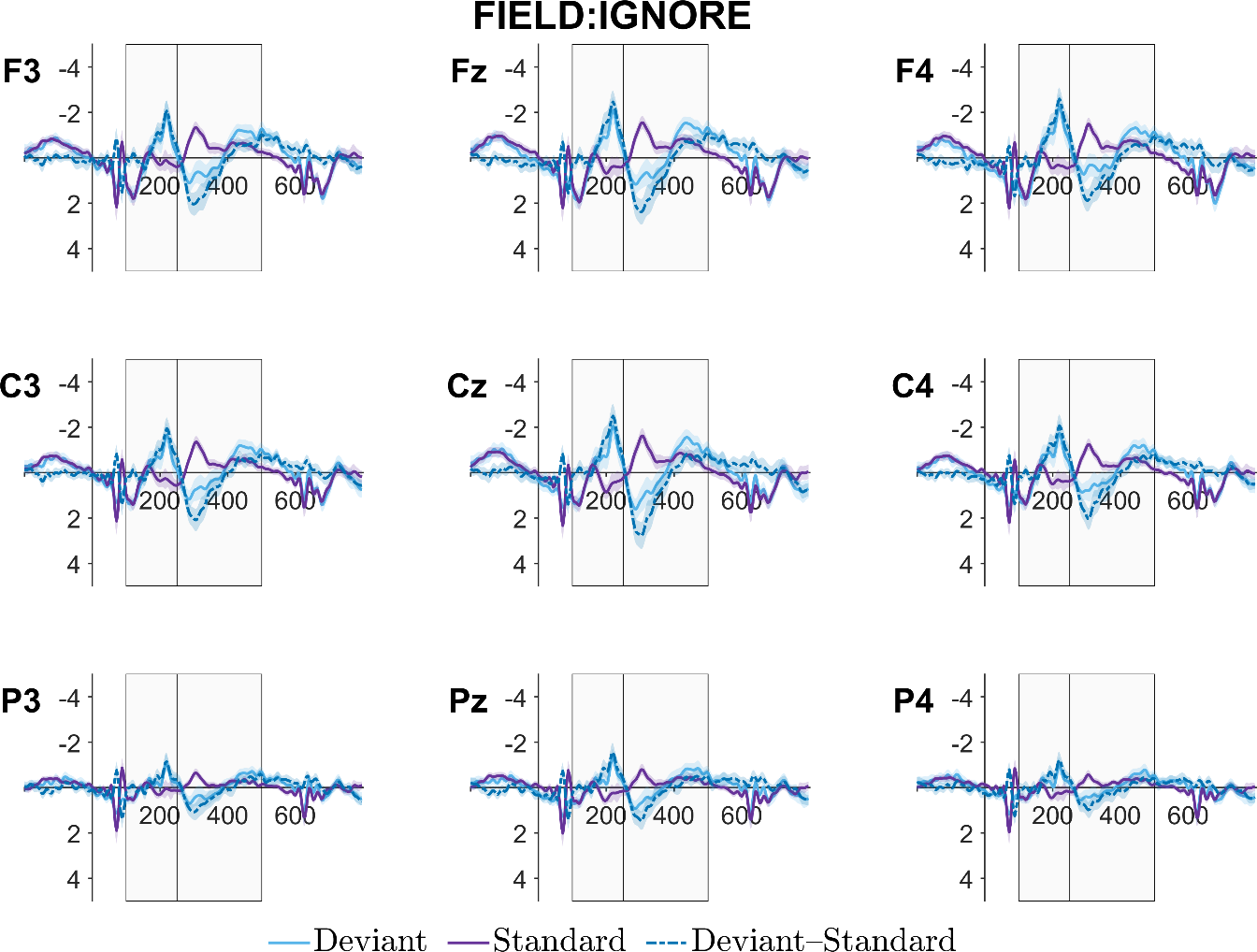
**

***Figure S3.*** Grand average event-related potentials (ERPs; solid lines) and difference waveforms (broken lines) from nine electrodes (F3, Fz, F4, C3, Cz, C4, P3, Pz, P4) during performance of the Ignore task in the Field condition. Light blue waveforms depict ERPs evoked by deviant stimuli; purple waveforms depict ERPs evoked by standard stimuli. Broken lines indicate the difference in mean voltage (*μV*) between deviants and standards. Shading indicates the 84% confidence interval.

**
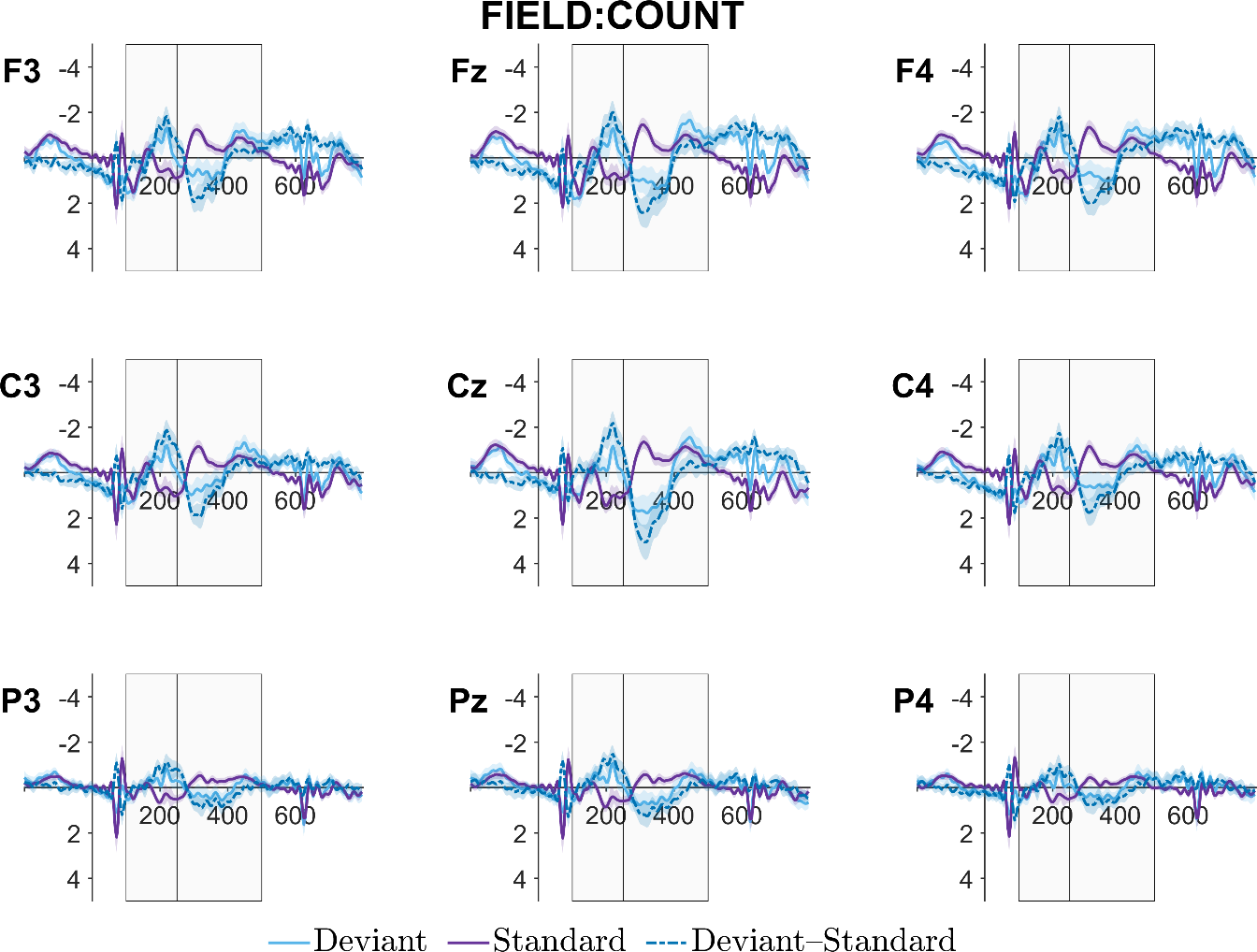
**

***Figure S4.*** Grand average event-related potentials (ERPs; solid lines) and difference waveforms (broken lines) from nine electrodes (F3, Fz, F4, C3, Cz, C4, P3, Pz, P4) during performance of the Count task in the Field condition. Light blue waveforms depict ERPs evoked by deviant stimuli; purple waveforms depict ERPs evoked by standard stimuli. Broken lines indicate the difference in mean voltage (*μV*) between deviants and standards. Shading indicates the 84% confidence interval.

**
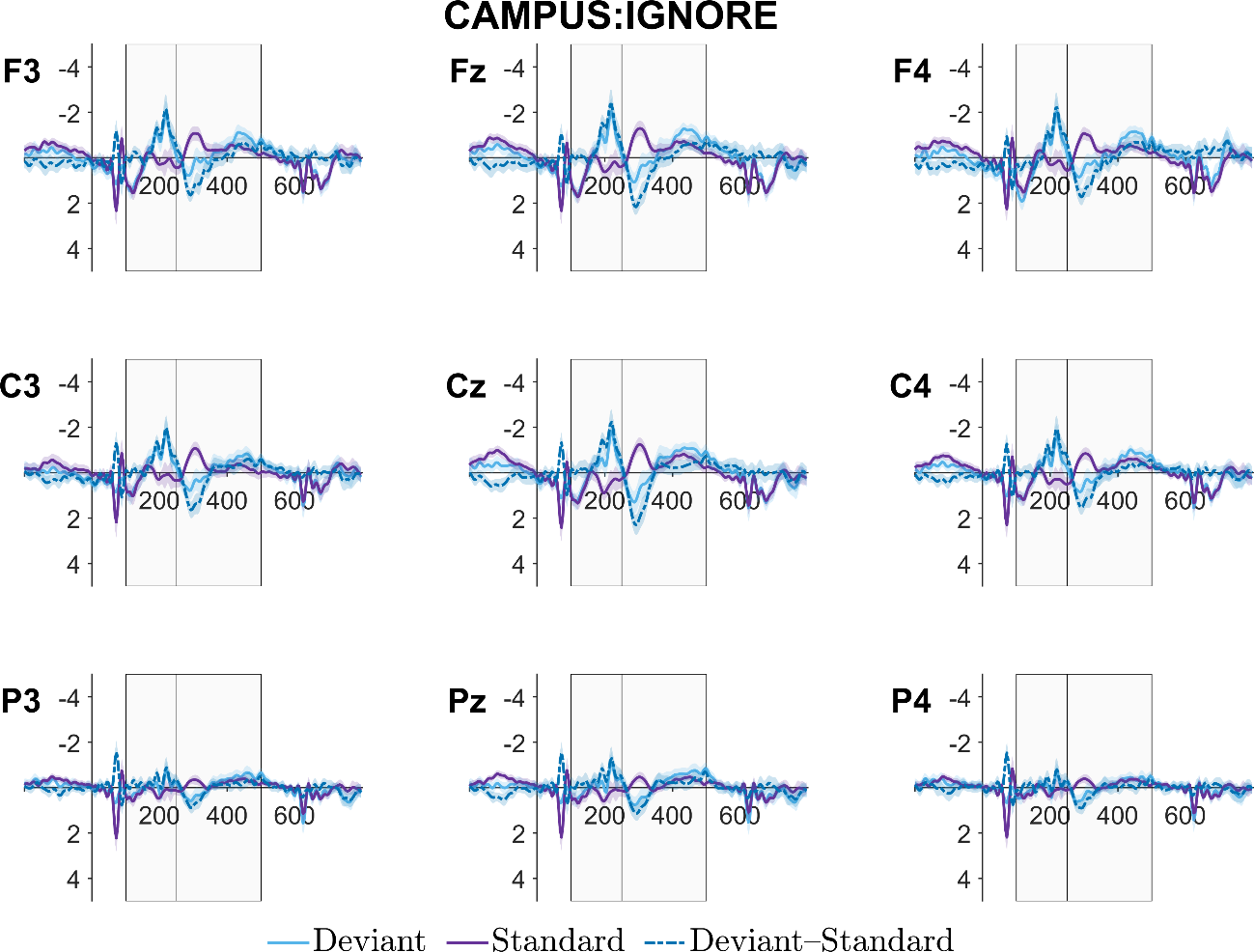
**

***Figure S5.*** Grand average event-related potentials (ERPs; solid lines) and difference waveforms (broken lines) from nine electrodes (F3, Fz, F4, C3, Cz, C4, P3, Pz, P4) during performance of the Ignore task in the Campus condition. Light blue waveforms depict ERPs evoked by deviant stimuli; purple waveforms depict ERPs evoked by standard stimuli. Broken lines indicate the difference in mean voltage (*μV*) between deviants and standards. Shading indicates the 84% confidence interval.


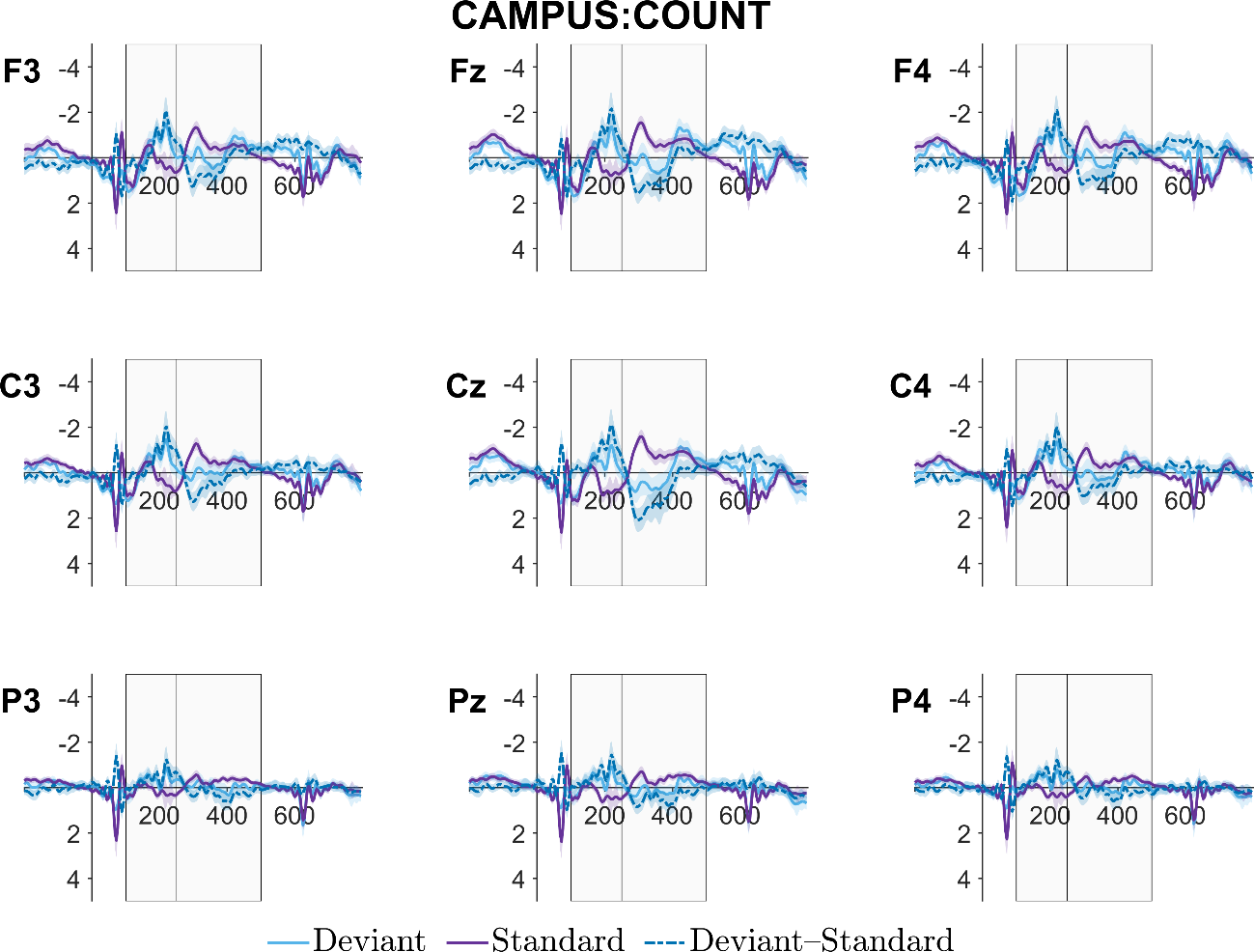


***Figure S6.*** Grand average event-related potentials (ERPs; solid lines) and difference waveforms (broken lines) from nine electrodes (F3, Fz, F4, C3, Cz, C4, P3, Pz, P4) during performance of the Count task in the Campus condition. Light blue waveforms depict ERPs evoked by deviant stimuli; purple waveforms depict ERPs evoked by standard stimuli. Broken lines indicate the difference in mean voltage (*μV*) between deviants and standards. Shading indicates the 84% confidence interval.

**Table S1**. Summary of linear mixed-effects models of ERP time-windows factorised by midline electrode channel.

|  | **MMN time-window** | | | | **P3 time-window** | | | |
| --- | --- | --- | --- | --- | --- | --- | --- | --- |
| *Predictors* | *Estimate* | *S.E.* | *t-stat* | *p-value* | *Estimate* | *S.E.* | *t-stat* | *p-value* |
| (Intercept) | 0.28 | 0.07 | 3.98 | **<0.001** | -0.23 | 0.06 | -3.56 | **0.001** |
| stimulus[S.Standard] | 0.28 | 0.01 | 30.11 | **<0.001** | -0.26 | 0.04 | -7.17 | **<0.001** |
| task[S.Ignore] | 0.00 | 0.01 | 0.13 | 0.898 | -0.07 | 0.01 | -9.38 | **<0.001** |
| environ[S.Lab] | 0.19 | 0.05 | 3.65 | **0.001** | -0.04 | 0.05 | -0.77 | 0.448 |
| environ[S.Field] | -0.10 | 0.03 | -3.04 | **0.004** | 0.04 | 0.03 | 1.18 | 0.246 |
| channel_id[S.Cz] | 0.12 | 0.02 | 7.41 | **<0.001** | -0.15 | 0.04 | -4.30 | **<0.001** |
| channel_id[S.Fz] | 0.20 | 0.02 | 12.31 | **<0.001** | -0.20 | 0.04 | -5.04 | **<0.001** |
| channel_id[S.Oz] | -0.17 | 0.02 | -10.40 | **<0.001** | 0.22 | 0.05 | 4.08 | **<0.001** |
| prestim.s | -1.39 | 0.01 | -200.96 | **<0.001** | -1.30 | 0.01 | -244.75 | **<0.001** |
| stimulus[S.Standard] : task[S.Ignore] | -0.01 | 0.01 | -1.03 | 0.301 | 0.07 | 0.01 | 9.78 | **<0.001** |
| stimulus[S.Standard] : environ[S.Lab] | 0.14 | 0.01 | 10.39 | **<0.001** | -0.17 | 0.01 | -16.80 | **<0.001** |
| stimulus[S.Standard] : environ[S.Field] | -0.05 | 0.01 | -3.53 | **<0.001** | 0.05 | 0.01 | 5.24 | **<0.001** |
| task[S.Ignore] : environ[S.Lab] | 0.04 | 0.01 | 3.23 | **0.001** | -0.10 | 0.01 | -9.42 | **<0.001** |
| task[S.Ignore] : environ[S.Field] | -0.06 | 0.01 | -4.89 | **<0.001** | 0.01 | 0.01 | 0.89 | 0.375 |
| stimulus[S.Standard] : channel_id[S.Cz] | 0.14 | 0.02 | 9.08 | **<0.001** | -0.17 | 0.01 | -13.91 | **<0.001** |
| stimulus[S.Standard] : channel_id[S.Fz] | 0.02 | 0.02 | 1.24 | 0.214 | -0.09 | 0.01 | -7.62 | **<0.001** |
| stimulus[S.Standard] : channel_id[S.Oz] | -0.21 | 0.02 | -12.42 | **<0.001** | 0.23 | 0.01 | 17.82 | **<0.001** |
| task[S.Ignore] : channel_id[S.Cz] | -0.02 | 0.02 | -1.35 | 0.177 | -0.04 | 0.01 | -3.13 | **0.002** |
| task[S.Ignore] : channel_id[S.Fz] | -0.03 | 0.02 | -2.13 | **0.034** | 0.01 | 0.01 | 0.73 | 0.468 |
| task[S.Ignore] : channel_id[S.Oz] | 0.00 | 0.02 | 0.22 | 0.825 | 0.06 | 0.01 | 5.08 | **<0.001** |
| environ[S.Lab] : channel_id[S.Cz] | 0.14 | 0.02 | 6.31 | **<0.001** | -0.03 | 0.02 | -1.74 | 0.081 |
| environ[S.Field] : channel_id[S.Cz] | -0.07 | 0.02 | -3.14 | **0.002** | 0.03 | 0.02 | 1.75 | 0.080 |
| environ[S.Lab] : channel_id[S.Fz] | 0.17 | 0.02 | 7.52 | **<0.001** | -0.06 | 0.02 | -3.59 | **<0.001** |
| environ[S.Field] : channel_id[S.Fz] | -0.06 | 0.02 | -2.73 | **0.006** | 0.02 | 0.02 | 1.42 | 0.156 |
| environ[S.Lab] : channel_id[S.Oz] | -0.19 | 0.02 | -8.29 | **<0.001** | 0.02 | 0.02 | 1.05 | 0.292 |
| environ[S.Field] : channel_id[S.Oz] | 0.09 | 0.02 | 3.79 | **<0.001** | -0.01 | 0.02 | -0.49 | 0.623 |
| stimulus[S.Standard] : task[S.Ignore] : environ[S.Lab] | -0.01 | 0.01 | -0.79 | 0.429 | 0.04 | 0.01 | 3.84 | **<0.001** |
| stimulus[S.Standard] : task[S.Ignore] : environ[S.Field] | 0.03 | 0.01 | 1.93 | 0.054 | -0.01 | 0.01 | -1.38 | 0.168 |
| stimulus[S.Standard] : task[S.Ignore] : channel_id[S.Cz] | 0.01 | 0.02 | 0.35 | 0.724 | 0.05 | 0.01 | 3.84 | **<0.001** |
| stimulus[S.Standard] : task[S.Ignore] : channel_id[S.Fz] | 0.09 | 0.02 | 5.66 | **<0.001** | -0.03 | 0.01 | -2.39 | **0.017** |
| stimulus[S.Standard] : task[S.Ignore] : channel_id[S.Oz] | -0.03 | 0.02 | -1.59 | 0.111 | -0.05 | 0.01 | -3.99 | **<0.001** |
| stimulus[S.Standard] : environ[S.Lab] : channel_id[S.Cz] | 0.02 | 0.02 | 0.96 | 0.339 | -0.05 | 0.02 | -3.14 | **0.002** |
| stimulus[S.Standard] : environ[S.Field] : channel_id[S.Cz] | -0.00 | 0.02 | -0.10 | 0.921 | 0.01 | 0.02 | 0.33 | 0.738 |
| stimulus[S.Standard] : environ[S.Lab] : channel_id[S.Fz] | -0.03 | 0.02 | -1.39 | 0.164 | -0.03 | 0.02 | -1.50 | 0.134 |
| stimulus[S.Standard] : environ[S.Field] : channel_id[S.Fz] | 0.01 | 0.02 | 0.50 | 0.616 | -0.00 | 0.02 | -0.14 | 0.885 |
| stimulus[S.Standard] : environ[S.Lab] : channel_id[S.Oz] | -0.05 | 0.02 | -2.29 | **0.022** | 0.11 | 0.02 | 6.23 | **<0.001** |
| stimulus[S.Standard] : environ[S.Field] : channel_id[S.Oz] | 0.02 | 0.02 | 0.75 | 0.452 | -0.03 | 0.02 | -1.44 | 0.151 |
| task[S.Ignore] : environ[S.Lab] : channel_id[S.Cz] | 0.02 | 0.02 | 0.74 | 0.462 | 0.01 | 0.02 | 0.32 | 0.752 |
| task[S.Ignore] : environ[S.Field] : channel_id[S.Cz] | -0.03 | 0.02 | -1.53 | 0.127 | -0.02 | 0.02 | -1.00 | 0.318 |
| task[S.Ignore] : environ[S.Lab] : channel_id[S.Fz] | -0.03 | 0.02 | -1.50 | 0.134 | 0.05 | 0.02 | 2.92 | **0.003** |
| task[S.Ignore] : environ[S.Field] : channel_id[S.Fz] | 0.03 | 0.02 | 1.39 | 0.165 | -0.05 | 0.02 | -3.10 | **0.002** |
| task[S.Ignore] : environ[S.Lab] : channel_id[S.Oz] | -0.01 | 0.02 | -0.27 | 0.786 | -0.01 | 0.02 | -0.58 | 0.560 |
| task[S.Ignore] : environ[S.Field] : channel_id[S.Oz] | 0.00 | 0.02 | 0.09 | 0.929 | 0.04 | 0.02 | 2.25 | **0.025** |
| stimulus[S.Standard] : task[S.Ignore] : environ[S.Lab] : channel_id[S.Cz] | 0.01 | 0.02 | 0.61 | 0.542 | -0.01 | 0.02 | -0.64 | 0.523 |
| stimulus[S.Standard] : task[S.Ignore] : environ[S.Field] : channel_id[S.Cz] | -0.01 | 0.02 | -0.57 | 0.567 | 0.02 | 0.02 | 1.14 | 0.254 |
| stimulus[S.Standard] : task[S.Ignore] : environ[S.Lab] : channel_id[S.Fz] | 0.03 | 0.02 | 1.39 | 0.166 | -0.04 | 0.02 | -2.31 | **0.021** |
| stimulus[S.Standard] : task[S.Ignore] : environ[S.Field] : channel_id[S.Fz] | -0.02 | 0.02 | -0.78 | 0.433 | 0.04 | 0.02 | 2.40 | **0.017** |
| stimulus[S.Standard] : task[S.Ignore] : environ[S.Lab] : channel_id[S.Oz] | -0.01 | 0.02 | -0.59 | 0.554 | 0.02 | 0.02 | 0.89 | 0.374 |
| stimulus[S.Standard] : task[S.Ignore] : environ[S.Field] : channel_id[S.Oz] | 0.04 | 0.02 | 1.49 | 0.137 | -0.04 | 0.02 | -2.43 | **0.015** |
| **Random Effects** | | | | | | | | |
| σ^2^ | 31.70 | | | | 18.60 | | | |
| τ_00_ | 0.17 _subject_id_ | | | | 0.15 _subject_id_ | | | |
| τ_11_ | 0.09 _subject_id.environ[S.Lab]_ | | | | 0.04 _subject_id.stimulus[S.Standard]_ | | | |
|  | 0.03 _subject_id.environ[S.Field]_ | | | | 0.09 _subject_id.environ[S.Lab]_ | | | |
|  |  | | | | 0.04 _subject_id.environ[S.Field]_ | | | |
|  |  | | | | 0.04 _subject_id.channel_id[S.Cz]_ | | | |
|  |  | | | | 0.05 _subject_id.channel_id[S.Fz]_ | | | |
|  |  | | | | 0.10 _subject_id.channel_id[S.Oz]_ | | | |
| ρ_01_ | 0.05 _subject_id.environ[S.Lab]_ | | | | -0.19 _subject_id.stimulus[S.Standard]_ | | | |
|  | 0.12 _subject_id.environ[S.Field]_ | | | | 0.20 _subject_id.environ[S.Lab]_ | | | |
|  |  | | | | 0.03 _subject_id.environ[S.Field]_ | | | |
|  |  | | | | 0.70 _subject_id.channel_id[S.Cz]_ | | | |
|  |  | | | | 0.74 _subject_id.channel_id[S.Fz]_ | | | |
|  |  | | | | -0.83 _subject_id.channel_id[S.Oz]_ | | | |
| N | 36 _subject_id_ | | | | 36 _subject_id_ | | | |
| Observations | 572291 | | | | 572291 | | | |

*Note*. Factor variables sum-to-zero contrast-coded. *Estimate* = beta coefficient, *S.E.* = standard error, *stimulus* = oddball task stimulus (Deviant, Standard), *task* = cognitive task condition (Count, Ignore), *environ* = environmental context (Lab, Field, Campus), *channel_id* = electrode channel location (Fz, Cz, Pz, Oz), *prestim.s* = prestimulus EEG voltage (scaled), σ^2^ = residual variance, τ_00_ = random intercept variance, τ_11_ = random slope variance, ρ_01_ = random effect correlation coefficient, *N* = number of levels per grouping variable.

**
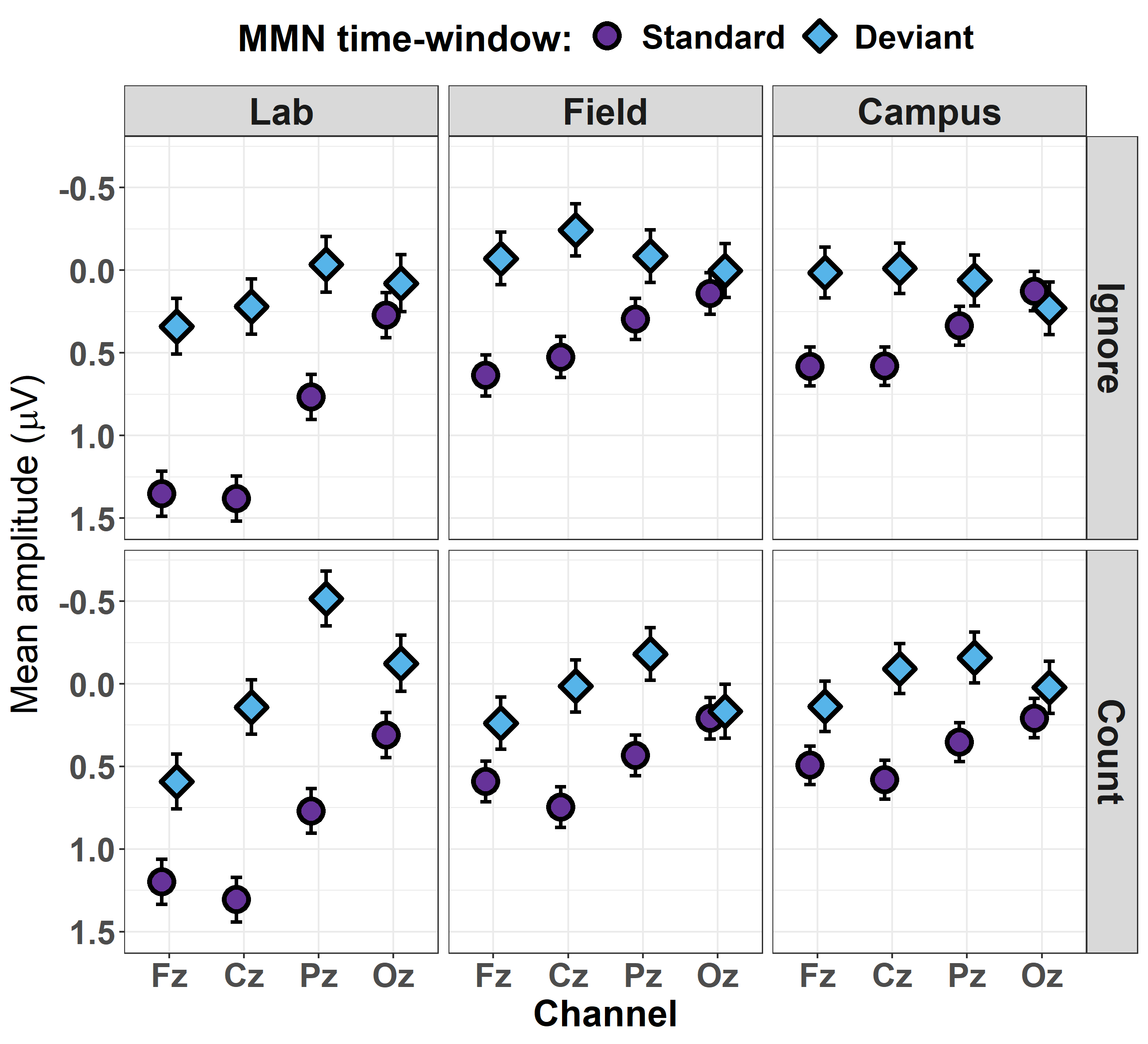
**

***Figure S7.*** Visualisation of estimated marginal mean voltages for the MMN time-window (100-250 ms) factorized by stimulus (Standard, Deviant), task (Ignore, Count), environment (Lab, Field, Campus), and channel location (Fz, Cz, Pz, Oz). Error bars indicate 84% confidence intervals.

**
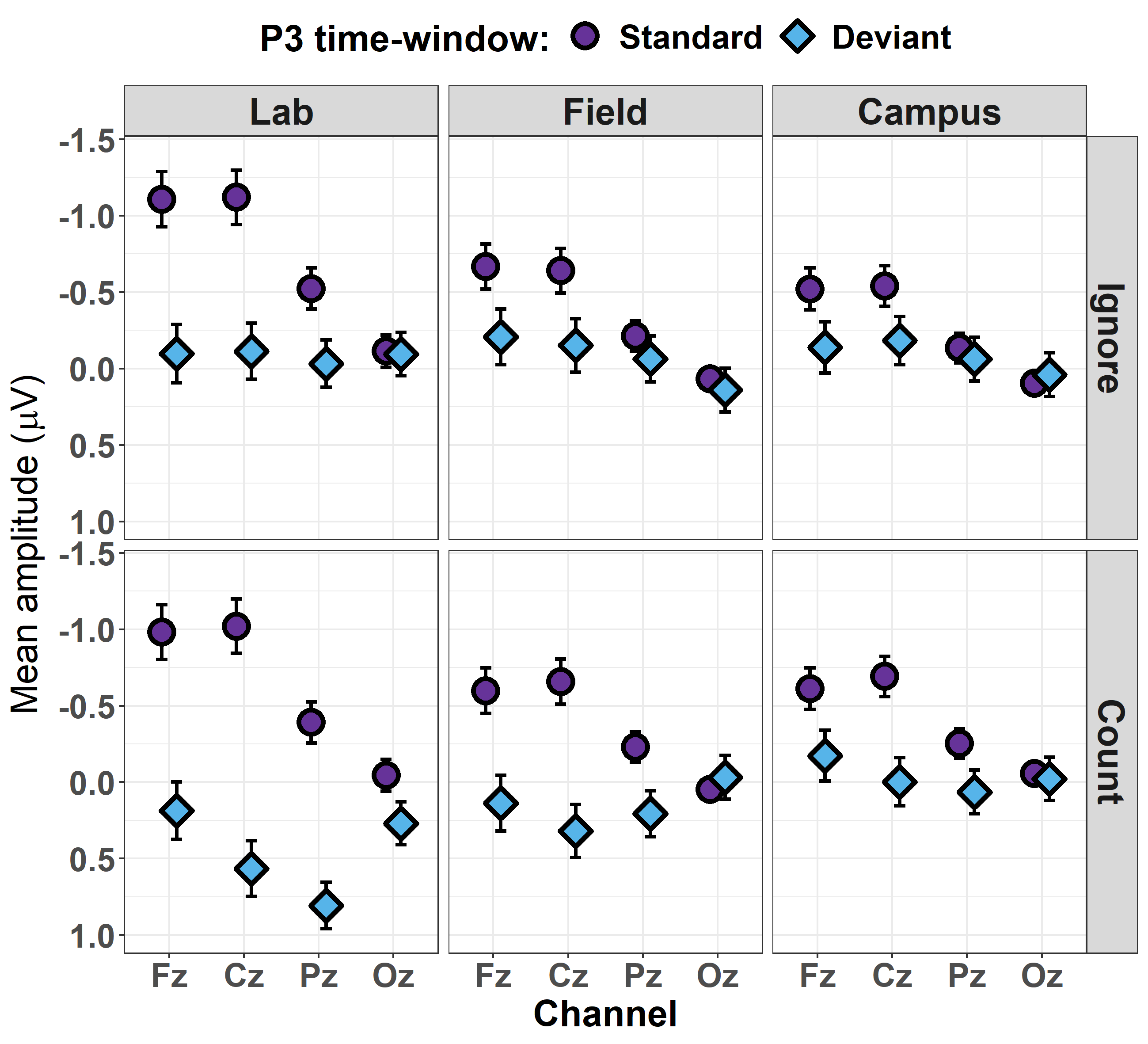
**

***Figure S8***. Visualisation of estimated marginal mean voltages for the P3 time-window (250-500 ms) factorized by stimulus (Standard, Deviant), task (Ignore, Count), environment (Lab, Field, Campus), and channel location (Fz, Cz, Pz, Oz). Error bars indicate 84% confidence intervals.
